# Supplementary material for: A power approximation for the Kenward and Roger Wald test in the linear mixed model
Source: PLoS One. 2021 Jul 21;16(7):e0254811. doi: 10.1371/journal.pone.0254811 (PMC8294572; doi:10.1371/journal.pone.0254811)
Supplement: Attachment — Submitted filename: Review_Report.pdf [file pone.0254811.s001.pdf]

A Power Approximation for the Kenward and Roger Wald Test in the Linear Mixed Model

In this paper, a power approximation of Kenward and Roger test is derived. Via Monte Carlo simulation, author's demonstrate that the new power approximation is accurate for cluster randomized trials and longitudinal study designs.

The paper is well written and addresses an interesting problem. My major issues are listed below.

Major comments:

Comment 1: On line 171, it is claimed that if Σ_s and β are estimated using multivariate techniques, independence would follow. Provide a reference for this or give a detailed explanation in support of this claim.

Comment 2: The comparison of empirical and proposed powers is done assuming intraclass correlation (ICC) 0.04. This value of ICC is very small and in practice it can vary up to 0.5. Make the comparison of powers for higher values of ICC as well (say 0.1, 0.2, 0.5).

Comment 3: In the spirit of the longitudinal studies, how efficient is the power approximation when the correlation structure is assumed to be auto–regressive?

Comment 4: In Section 5 (Applied Example), rather than assuming the values of standard deviation and intraclass correlation, it is more reasonable to use the estimates of the parameters obtained from the data.

Minor comments:

Comment 1: Check line 122.
