# Supplementary material for: A power approximation for the Kenward and Roger Wald test in the linear mixed model
Source: PLoS One. 2021 Jul 21;16(7):e0254811. doi: 10.1371/journal.pone.0254811 (PMC8294572; doi:10.1371/journal.pone.0254811)
Supplement: Attachment — Submitted filename: Repsonse to Editor Requests 04.pdf [file pone.0254811.s003.pdf]

Response to Editor Requests

We thank the editor for their careful review. We have responded to all requests below. Editor comments are in italics and our response is in plain type.

Requests

1. *We note that your manuscript is not formatted using one of PLOS ONE's accepted file types. Please reattach your manuscript as one of the following file types: .doc, .docx, .rtf, or .tex (accompanied by a .pdf). If your submission was prepared in LaTex, please submit your manuscript file in PDF format and attach your .tex file as "other."*

We now include the .tex file, along with the PDF, as requested. We apologize for missing this requirement in the earlier submission.

2. *Thanks for clarifying the timing of Dr. Kreidler's affiliation with Sunrun as follows:*

"We have removed Dr. Kreidler's affiliation with SunRun, Inc. Previously we indicated that Dr. Kreidler was affiliated with SunRun Inc. After further review, and examining the policies of PLOS One, we realized that SunRun did not play any role in the study design, data collection and analysis, decision to publish, or preparation of the manuscript and, in addition, did not provide support in the form of salaries for any author, including SMK. We have updated Dr. Kreidler's affiliation to be the Department of Biostatistics and Information, University of Colorado Denver. Although Dr. Kreidler is currently employed by SunRun, Inc., all of the work on this manuscript was completed while Dr. Kreidler was a doctoral student at the University of Colorado Denver. Current revisions are being done during Dr. Kreidler's personal time. No SunRun, Inc. resources were used in the revisions of this manuscript for submission. The publication of this manuscript will not affect SunRun Inc., nor Dr. Kreidler in any financial way."

Thank you also for proposing the following Financial Disclosure statement:

""KEM, DHG - NIDCR 1 R01 DE020832-01A1 - The National Institute of Dental and Craniofacial Research - www.nih.gov - No KEM, DHG - NIGMS 9R01GM121081-05 - The National Institute of General Medical Sciences www.nih.gov- No DD - OD 5UG3OD023248-02 - Office of the Director - www.nih.gov No. The funders had no role in study design, data collection and analysis, decision to publish, or preparation of the manuscript."

We note your Competing Interests statement currently reads as follows:

"The authors have declared that no competing interests exist."

Please note that declaring all potential competing interests is a requirement at PLOS and is integral to the transparent reporting of research: https://journals.plos.org/plosone/s/competing-interests. A competing interest is anything that interferes with, or could reasonably be perceived as interfering with, the full and objective presentation, peer review, editorial decision-making, or publication of research or non-research articles submitted to PLOS. Competing interests can be financial or non-financial, professional, or personal. Competing interests can arise in relationship to an institution, organization, or another person, including employment with a commercial organization. Though Dr. Kreidler's affiliation with Sunrun began after the study was conducted, for full transparency, it will still need to be declared in your Competing Interests statement.

As such, we've drafted for your approval the following proposed Financial Disclosure and Competing Interests statements, which include all required declarations and adhere to the PLOS ONE formatting conventions:

"This study was supported by The National Institute of Dental and Craniofacial Research (www.nih.gov) in the form of a grant awarded to KEM and DHG (NIDCR 1 R01 DE020832-01A1), The National Institute of General Medical Sciences (www.nih.gov) in the form of a grant awarded to KEM and DHG (NIGMS 9R01GM121081-05), and the Office of the Director (www.nih.gov) in the form of a grant awarded to Dana Dabelea, PI (OD 5UG3OD023248-02). The funders had no role in study design, data collection and analysis, decision to publish, or preparation of the manuscript."

"The authors have read the journal's policy and have the following competing interests: SMK is an employee of Sunrun, Inc., but was not affiliated with the company at the time the study was conducted. This does not alter our adherence to PLOS ONE policies on sharing data and materials. There are no patents, products in development or marketed products associated with this research to declare."

Please confirm whether this information is accurate, and whether we may update your Financial Disclosure and Competing Interests statements accordingly on your behalf.

We confirm that the information is accurate. Thank you for drafting the correction. Please use your proposed language to update the Financial Disclosure and Competing Interests statements on our behalf.

3. *Please also remove all funding information from the Acknowledgments section of your manuscript.*

We have now removed all funding information from the Acknowledgements section of the manuscript. The Acknowledgements section now only reads as follows:

"A portion of this paper was submitted to the University of Colorado Denver in partial fulfillment of the requirements for the degree of Doctor of Philosophy in Biostatistics for Dr. Sarah M. Kreidler."
