# Supplementary material for: A power approximation for the Kenward and Roger Wald test in the linear mixed model
Source: PLoS One. 2021 Jul 21;16(7):e0254811. doi: 10.1371/journal.pone.0254811 (PMC8294572; doi:10.1371/journal.pone.0254811)
Supplement: Attachment — Submitted filename: Comments.docx [file pone.0254811.s002.docx]

The authors here present a noncentral F power approximation for the Kenward and Roger test. This work is innovative, and the organization of the manuscript is clear and comprehensive. Below are some minor comments that could help further streamline the text.

General comments:

1. The homoscedasticity assumption (line 136) the author made could be a key limitation of the approximation method with the presence of different random coefficients. Even though it has been mentioned that there are no repeated covariates (line 82), it is worth mentioning it in the Discussion section.

2. Theorem 3 in the Appendix lacks the reference regarding “the sum of the inverse Wishart distribution asymptotically or approximately follows an inverse Wishart distribution.”

Minor comments:

1. Using $p_{i}$ to denote the number of observations is counter-intuitive, especially given you define $n=\sum_{i=1}^{N} p_{i}$. Replacing $p_{i}$ with $n_{i}$ would be easier to read.

2. Duplicated terms appeared in line 122.

3. The notation for noncentrality parameter of F distribution is very similar to the notation of Wald statistic, please consider changing one of them.

4. Box-plot elements should be defined (e.g. center line, median; box limits, upper and lower quartiles; whiskers, 1.5x interquartile range; points, outliers) in the legend.
